# Endothelial dysfunction: molecular mechanisms and clinical implications

**DOI:** 10.1002/mco2.651

**Published:** 2024-07-22

**Authors:** Xia Wang, Ben He

**Affiliations:** ^1^ Department of Cardiology Shanghai Chest Hospital, Shanghai Jiao Tong University School of Medicine Shanghai China

**Keywords:** cardiovascular disease, endothelial dysfunction, inflammation, nitric oxide, oxidative stress

## Abstract

Cardiovascular disease (CVD) and its complications are a leading cause of death worldwide. Endothelial dysfunction plays a crucial role in the initiation and progression of CVD, serving as a pivotal factor in the pathogenesis of cardiovascular, metabolic, and other related diseases. The regulation of endothelial dysfunction is influenced by various risk factors and intricate signaling pathways, which vary depending on the specific disease context. Despite numerous research efforts aimed at elucidating the mechanisms underlying endothelial dysfunction, the precise molecular pathways involved remain incompletely understood. This review elucidates recent research findings on the pathophysiological mechanisms involved in endothelial dysfunction, including nitric oxide availability, oxidative stress, and inflammation‐mediated pathways. We also discuss the impact of endothelial dysfunction on various pathological conditions, including atherosclerosis, heart failure, diabetes, hypertension, chronic kidney disease, and neurodegenerative diseases. Furthermore, we summarize the traditional and novel potential biomarkers of endothelial dysfunction as well as pharmacological and nonpharmacological therapeutic strategies for endothelial protection and treatment for CVD and related complications. Consequently, this review is to improve understanding of emerging biomarkers and therapeutic approaches aimed at reducing the risk of developing CVD and associated complications, as well as mitigating endothelial dysfunction.

## Graphical abstract

Endothelial dysfunction is commonly linked to various disease states resulting from an imbalance in the production of vasodilators and vasoconstrictors, among other factors. Human diseases associated with endothelial dysfunction include atherosclerosis, diabetes, metabolic syndrome, neurodegenerative diseases, chronic kidney disease, hypertension, heart failure, and stroke.

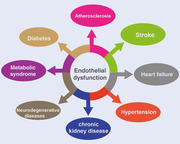

## 1 INTRODUCTION

The endothelium plays a crucial role in maintaining vascular homeostasis by regulating vascular tone, proliferation of vascular smooth muscle cells (VSMC), immune cell adhesion, and vascular inflammation via the production of bioactive factors.^1^ These factors include endothelium‐derived relaxing factors like prostaglandins and nitric oxide (NO), as well as endothelium‐derived contracting factors like angiotensin II (Ang II) and endothelin‐1 (ET‐1). The concept of compromised endothelial dysfunction is defined by diminished vasodilation or alterations impacting the vasoprotective homeostatic function.

Endothelial dysfunction serves as the predominant etiology of CVD, primarily stemming from decreased synthesis or efficacy of endothelium‐dependent hyperpolarization factors.^2^, ^3^, ^4^ Pathological risk factors, including smoking, hypoxia, hyperlipidemia, and diabetes, are able to induce endothelial dysfunction in the context of cardiovascular incidents. Monitoring of markers associated with endothelial dysfunction contributes to early diagnosis of disease. Acute‐phase proteins, cytokines (including IL‐6, IL‐1, and tumor necrosis factor‐alpha [TNF‐α]), adhesion molecules (intercellular adhesion molecule [ICAM] and vascular cell adhesion molecule [VCAM]), and endothelial microparticles (EMPs) have been extensively investigated as indicators of endothelial dysfunction.^5^, ^6^, ^7^ Furthermore, interventions targeting endothelial dysfunction, such as traditional medicine, engineered extracellular vesicles (EVs), and molecular techniques, have shown promise in the prevention and mitigation of cardiovascular events and their complications.^8^, ^9^, ^10^ Consequently, there is significant value in the exploration of novel biomarkers and therapeutic approaches for endothelial dysfunction.

The molecular mechanisms underlying endothelial dysfunction are complex and influenced by a multitude of pathological stimuli, including NO, low‐density lipoprotein (LDL), reactive oxygen species (ROS), shear stress, and high glucose.^11^, ^12^, ^13^, ^14^, ^15^ Although many researches regarding endothelial dysfunction have been studied, there still remains a deficiency in diagnostic and therapeutic strategies for endothelial dysfunction‐related diseases. Further elucidation of the molecular mechanisms governing endothelial dysfunction holds promise for advancing its clinical management.

This review examines recent research on endothelial dysfunction, focusing on its specific molecular mechanisms and role in pathological conditions. The challenges in current research on endothelial dysfunction are highlighted, providing a foundation for future studies. Specifically, our study gathered recent research on the signaling pathways involved in endothelial function to investigate its molecular mechanisms in diverse pathological states such as atherosclerosis, heart failure (HF), hypertension, diabetes, chronic kidney disease (CKD), and neurodegenerative disorders. Additionally, we synthesized current clinical practices, both pharmacological and nonpharmacological, for addressing endothelial dysfunction. Furthermore, we examined recent novel research discoveries and proposed potential avenues for future investigations into endothelial dysfunction.

## 2 MOLECULAR MECHANISMS OF ENDOTHELIAL DYSFUNCTION

The maintenance of vascular homeostasis is regulated by a healthy endothelium through the release of NO. Endothelial dysfunction, manifested by reduced NO production and sensitivity, plays an important role in disrupting vascular balance, leading to a proinflammatory, prothrombotic, and less compliant vessel wall.^16^ The involvement of ROS and heightened oxidative stress is crucial in the development of endothelial dysfunction, as ROS serve as significant second messengers in the signaling process within cells.^12^ The primary etiology of endothelial dysfunction is attributed to an imbalance between antioxidant defense mechanisms and ROS generation, resulting in vascular damage. Additionally, a compromised endothelium also exacerbates ROS production and amplifies vascular inflammation, indicating a correlation between oxidative stress, inflammation, and endothelial dysfunction.^17^, ^18^ Inflammatory stimuli, including proinflammatory cytokines and cardiovascular risk factors, activate proinflammatory signaling pathways in EC, leading to the upregulation of adhesion molecules. In the following sections, we will discuss molecular mechanisms of endothelial dysfunction in detail.

### 2.1 Impaired NO bioavailability and signaling pathways

**FIGURE 1:**
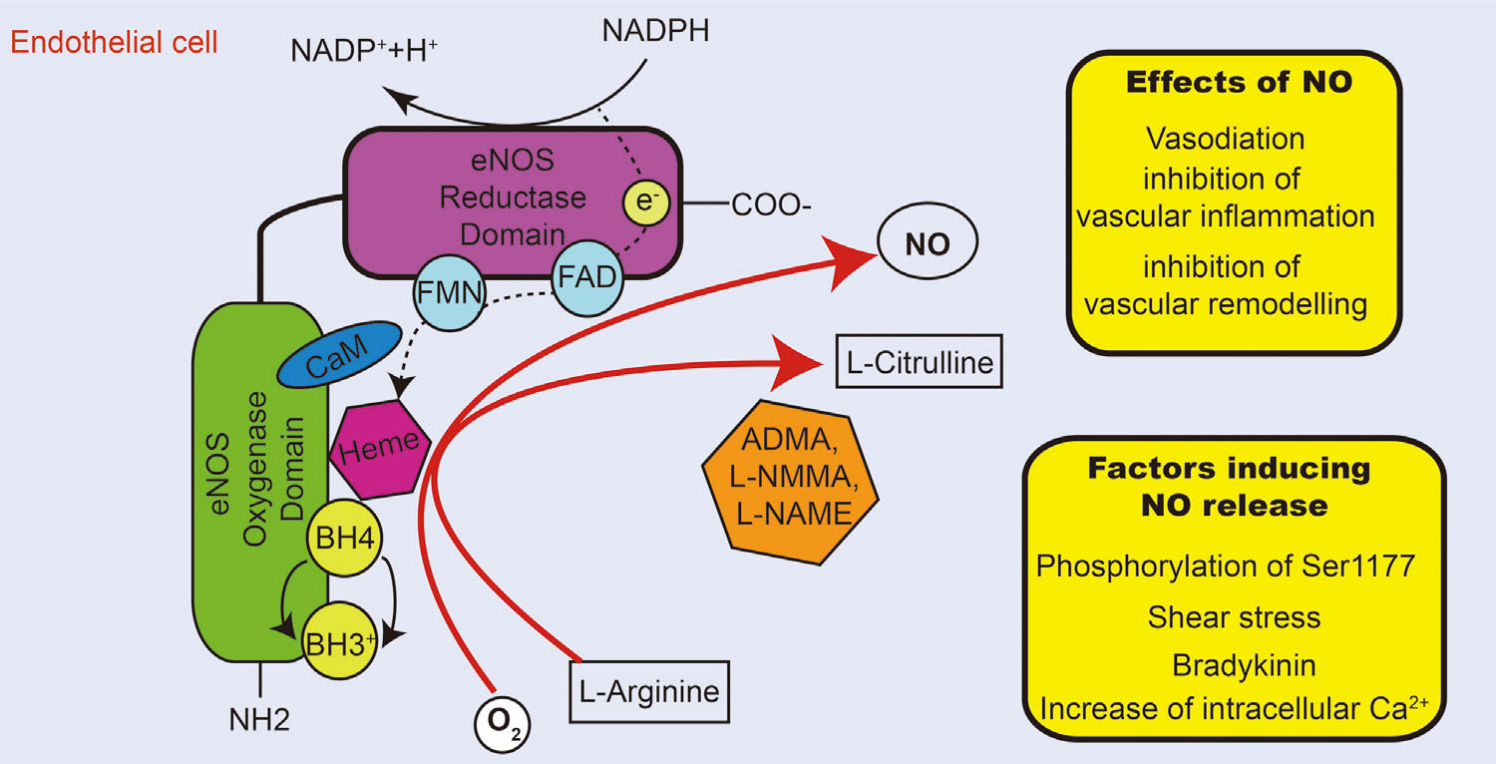
The generation of NO by eNOS coupling. By binding to the CaM, electrons are transferred through the FMN and FAD domains. In the presence of heme and BH4, NOS monomers form homodimers capable of catalyzing the transformation of l‐arginine and O_2_ into l‐citrulline and NO. *Abbreviations*: CaM, calcium signaling protein calmodulin; FMN, flavin adenine mononucleotide; FAD, flavin adenine dinucleotide.

NO is a gaseous free radical characterized by high activity and facile diffusion, synthesized by three distinct subtypes of NO synthase (NOS) enzymes: inducible NOS (iNOS), endothelial NOS (eNOS), and neuronal NOS (nNOS). The production of NO occurs through the catalysis of l‐arginine (l‐Arg) and O_2_ by NOS in EC, a process that necessitates the involvement of flavin adenine mononucleotide, flavin adenine dinucleotide, tetrahydrobiopterin (BH4), and dihydronicotinamide‐adenine dinucleotide phosphate‐II (NADPH‐II).^19^ The NOS enzyme facilitates the conversion of oxygen (O_2_) and l‐Arg to l‐citrulline and NO, utilizing electrons from NADPH. This enzymatic process is tightly regulated by various key proteins, prosthetic groups, and cofactors. In the presence of heme and BH4, NOS monomers form homo‐dimers that utilize NADPH electrons to catalyze the oxidation of l‐Arg to l‐citrulline and NO. This process involves the hydroxylation of l‐Arg to Nω‐hydroxy‐l‐arginine by NOS while the substrate is still bound to the enzyme. Subsequently, NOS catalyzes the oxidation of Nω‐hydroxy‐l‐arginine to l‐citrulline, leading to the release of NO (Figure 1).

The eNOS is predominantly expressed in ECs, as well as in various other cells such as cardiomyocyte, platelets, specific neurons, placental cells, and renal tubular epithelium.^19^ Within the endothelium, eNOS plays a crucial role in regulating blood flow and maintaining endothelial integrity, with its activity being modulated by intracellular calcium‐activated calmodulin (CaM). NOS are also regulated by a variety of posttranslational modifications, such as nitrosylation, phosphorylation, acetylation, glycosylation, and glutathionylation at multiple sites.^20^, ^21^, ^22^ Among these modifications, phosphorylation of Ser1177 residues in the reductase domain of eNOS is extensively studied, as it enhances electron flow and calcium sensitivity.^23^, ^24^, ^25^

**FIGURE 2:**
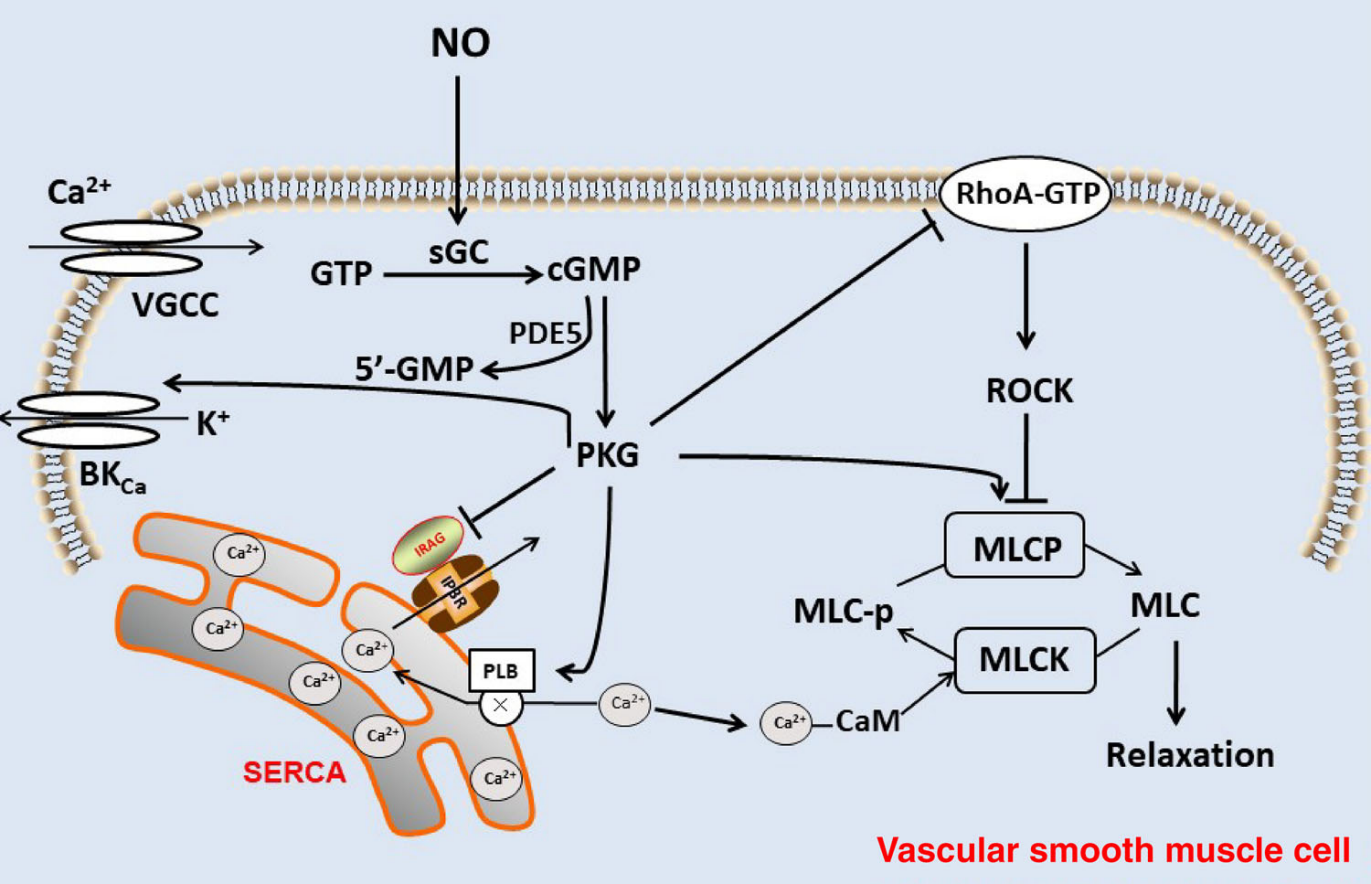
The NO signaling in VSMC. NO diffuses into adjacent VSMC and activates sGC, converting GTP to cGMP, which can be degraded by PDE5 to the inactive 5'‐GMP. cGMP causes vasodilation through PKG. *Abbreviations*: sGC, soluble guanylate cyclase; GTP, guanosine triphosphate; cGMP, cyclic guanosine monophosphate; PDE5, phosphodiesterase type 5; PKG, cGMP‐dependent protein kinase.

The diffusion and transduction of the NO signal occur from the production site to target cells, which can be the same cell, neighboring cells, or surrounding cells. Due to uncharged nature and partial lipophilicity of NO, it can freely cross the cell membrane. One of the main mechanisms involves PKG stimulating large conductance calcium‐activated potassium channels (BK_Ca_), which promotes potassium outflow, induces hyperpolarization, and reduces calcium influx. NO diffuses into neighboring VSMC and activates soluble guanylate cyclase (sGC), converting guanosine triphosphate (GTP) to cyclic guanosine monophosphate (cGMP), which can be degraded by phosphodiesterase type 5 (PDE5) to the inactive 5'‐GMP. cGMP causes vasodilation through cGMP‐dependent protein kinase (PKG).^26^ The main mechanisms are as follows (Figure 2): (1) PKG stimulates large conductance calcium to activate potassium channels (BK_Ca_), promotes K ^+^ outflow to induce hyperpolarization, and reduce Ca^2 +^influx; (2) inhibits the release of Ca^2+^ from sarcoplasmic reticulum to the cytoplasm via the IP_3_ receptor by repressing IP_3_ receptor‐associated PGG‐I substrate; (3) stimulates uptake of Ca^2+^ by the sarco/endoplasmic reticulum Ca2^2+^‐ATPase (SERCA) through phosphorylation of phospholamban (PLB), resulting in decrease of intracellular Ca^2+^ and inactivation of myosin light chain kinase (MLCK) by inhibiting the binding of Ca^2+^ and CaM, to inhibit myosin light chain (MLC) phosphorylation, which attenuates vasoconstriction; (4) dephosphorylates MLC by stimulating myosin light chain phosphatase (MLCP); (5) inhibits the activity of Rho A and resists the inhibitory effect of Rho‐associated protein kinase (ROCK) on MLCP, promotes the dephosphorylation of MLC, and inhibits the contractile effect, thus causes vasodilation. The NO–sGC–cGMP signaling pathway is known to play a crucial role in cardiovascular, renal, and metabolic functions. Specifically, endothelial‐derived NO is essential for regulating systemic blood pressure. Studies have shown that partial eNOS‐deficient mice exhibit blood–brain barrier (BBB) leakage and cerebral hypoperfusion at a young age, with worsening symptoms as they age.^27^ Additionally, eNOS has been found to have a beneficial effect in hypertrophic cardiomyopathy by protecting the heart against cardiac remodeling and fibrosis.^28^

The maintenance of vascular function by endothelial NO is essential under physiological conditions. Various interventions have been developed to enhance NO production. For instance, overexpression of Nur77 has been shown to mitigate endothelial dysfunction by promoting NO production and activating antioxidant pathways.^29^ Additionally, administration of inorganic nitrate has been found to prevent periodontitis‐induced endothelial dysfunction by restoring nitrite levels and subsequently NO levels.^30^ Furthermore, NO‐releasing glycosaminoglycans have been shown to expedite wound closure and reduce bacterial burden.^31^ Despite research has focused on studying the biology of NO and developing new tools to increase the biological activity of NO‐related signaling molecules in various diseases, especially in the cardiovascular system.^32^ The scope of approved clinical applications remains restricted. Although supplementation with l‐Arg, l‐citrulline, or BH4 has been shown to augment endogenous NOS activity, clinical trials for CVD have yielded conflicting outcomes, thereby underscoring the insufficiency of evidence supporting l‐Arg substitution.^33^

Furthermore, supplementation with the BH4 precursor folate or sapropterin has been examined in limited clinical trials, yielding inconclusive results. Larger clinical trials are necessary to further investigate the potential advantages of BH4 analogues.^34^ Nitroglycerin, also referred to as trinitroglycerin, is frequently employed in the treatment of acute myocardial infarction, angina pectoris, and severe hypertension, predating the discovery of the physiological role of NO.^35^ Long‐term use of organic nitrates may pose a risk of adverse effects such as endothelial dysfunction and hypotension, thereby limiting their therapeutic use.^36^ Thus, effectiveness of NO‐based treatments depends largely on the type of disease and the dose of NO used.

### 2.2 Oxidative stress and its effects on endothelial function

Oxidative stress in EC can result from an overabundance of free radical reactive substances, including ROS, active sulfur substances, and nitrogen reactive substances (RNS).^12^ Various factors such as oxLDL,^37^ homocysteine,^38^ aging,^39^ uric acid,^40^ high glucose, free fatty acids,^41^ Ang‐II^42^ can induce oxidative stress in EC. The generation of ROS primarily stems from NADPH oxidase, dysfunctional mitochondria, xanthine oxidase, and eNOS uncoupling.^43^ Excessive ROS can lead to the oxidation of macromolecules, including lipids, nucleic acids, and proteins. Moreover, antioxidant systems such as catalase, peroxidoredoxin, superoxide dismutase, glutathione peroxidase, and thioredoxin play a crucial role in regulating the oxidation process to maintain vascular homeostasis.^17^, ^44^, ^45^ Imbalance in ROS production, caused by either an increase in ROS‐producing systems or deficiencies in antioxidant enzymes, can lead to oxidative stress and potentially result in the development of diseases.

Recent research has demonstrated that Ang II triggers endothelial dysfunction and oxidative stress in mouse ophthalmic arteries through the activation of the Ang II type 1 receptor (AT1R) and the formation of NOX2‐dependent ROS.^46^ The buildup of RNS leading to nitrosative stress (NSS) and abnormal S‐nitrosylation of proteins, disrupting ER signaling, contributes to endothelial dysfunction induced by oxysterols and oxLDL.^47^ Conversely, the mitochondrial deacetylase Sirt3 mitigates endothelial dysfunction, vascular oxidative stress, vascular permeability, and reduces Ang II‐induced hypertension in mice.^48^ FOXO1 knockdown attenuates high glucose‐induced cellular senescence persistent and DNA damage by promoting MRN (MRE11–RAD50–NBS1 complex)–ATM pathway mediated DNA repair in HUVECs.

The efficacy of antioxidants in certain diseases has been documented in various studies. Research has examined the impact of current antioxidant medications, as well as the development of new anti‐oxidative stress drugs.^49^ Numerous clinical trials and meta‐analyses conducted over the last two decades have assessed the potential benefits of antioxidants such as A‐lipoic acid, vitamin C, vitamin E, xanthine oxidase inhibitors, and synthetic drugs like NXY‐059.^50^, ^51^, ^52^, ^53^ Despite these investigations, significant protective effects have not been consistently observed in large‐scale clinical trials. Recently, dapagliflozin was discovered to mitigate oxidative stress‐induced endothelial dysfunction by activation of sirtuin 1 in HUVEC.^54^ Similarly, roxadustat was found to mitigate Ang II‐induced hypertension, prevent cardiac hypertrophy, vascular thickening, and kidney injury by stabilizing HIF1α and targeting eNOS, AT1R, AT2R, and oxidative stress in mice.^55^ Furthermore, EVs, particularly engineered EVs, exhibited antioxidant properties. For example, small EVs derived from mesenchymal stem cells have been shown to diminish senescent biomarkers and restore angiogenesis and other dysfunctions in senescent EC stimulated by oxidative stress in vitro.^56^ Additionally, exosomes from angiotensin‐converting enzyme 2 (ACE2)‐enriched endothelial progenitor cells (EPC) have been found to suppress cellular senescence, oxidative stress, apoptosis, and endothelial dysfunction through the activation of the miR‐17‐5p/PTEN/PI3K/Akt signaling pathway, ultimately ameliorating cerebrovascular injury in elderly ischemic stroke mice.^57^ Although the role of oxidative stress in endothelial dysfunction is well understood, further exploration of targets that regulate ROS and mitigate oxidative stress is necessary to design new antioxidant therapies.

Given the important role of oxidative stress in endothelial dysfunction, identifying novel markers of oxidative stress (e.g., oxidation‐modified lipids and proteins) may be a promising strategy for preventing endothelial dysfunction‐related diseases. Endothelial oxidative stress (high plasma peroxide level) resulting in decreased NO bioavailability (5‐α‐nitroso hemoglobin level) may be a pathogenic factor for endothelial dysfunction in patients infected with COVID‐19.^58^ A cross‐sectional study on 100 patients with CKD on peritoneal dialysis, of whom 48 were diabetic patients and 52 patients were nondiabetic showed that ferric reducing ability of plasma and malondialdehyde (MDA) increased in blood samples of diabetic patients compared with that of nondiabetics CKD patients.^59^ A clinic study involved 164 rheumatoid arthritis patients and 100 age and sex matched healthy controls showed that levels of oxidative stress markers, MDA, and paroxonase‐1 (PON‐1) are negative correlation with peripheral vasodilation and the presence of endothelial dysfunction.^60^ These studies suggest that detection of oxidative stress‐related indicators may be a promising approach for early diagnosis of endothelial dysfunction‐related diseases.

### 2.3 Inflammation‐mediated pathways contributing to endothelial dysfunction

Vascular inflammation is believed to be a contributing factor to the development of CVD and other related conditions.^18^ In response to injury, EC release various inflammatory mediators such as chemokines, colony‐stimulating factors, IL‐8, interferon, and monocyte chemokine protein‐1 (MCP‐1). This leads to the adhesion of monocytes and neutrophils to the walls of arteries.^61^ Conversely, anti‐inflammatory cytokines such as IL‐35 and IL‐10 have been shown to reduce mitochondrial ROS production, thereby inhibiting endothelial activation.^62^

**FIGURE 3:**
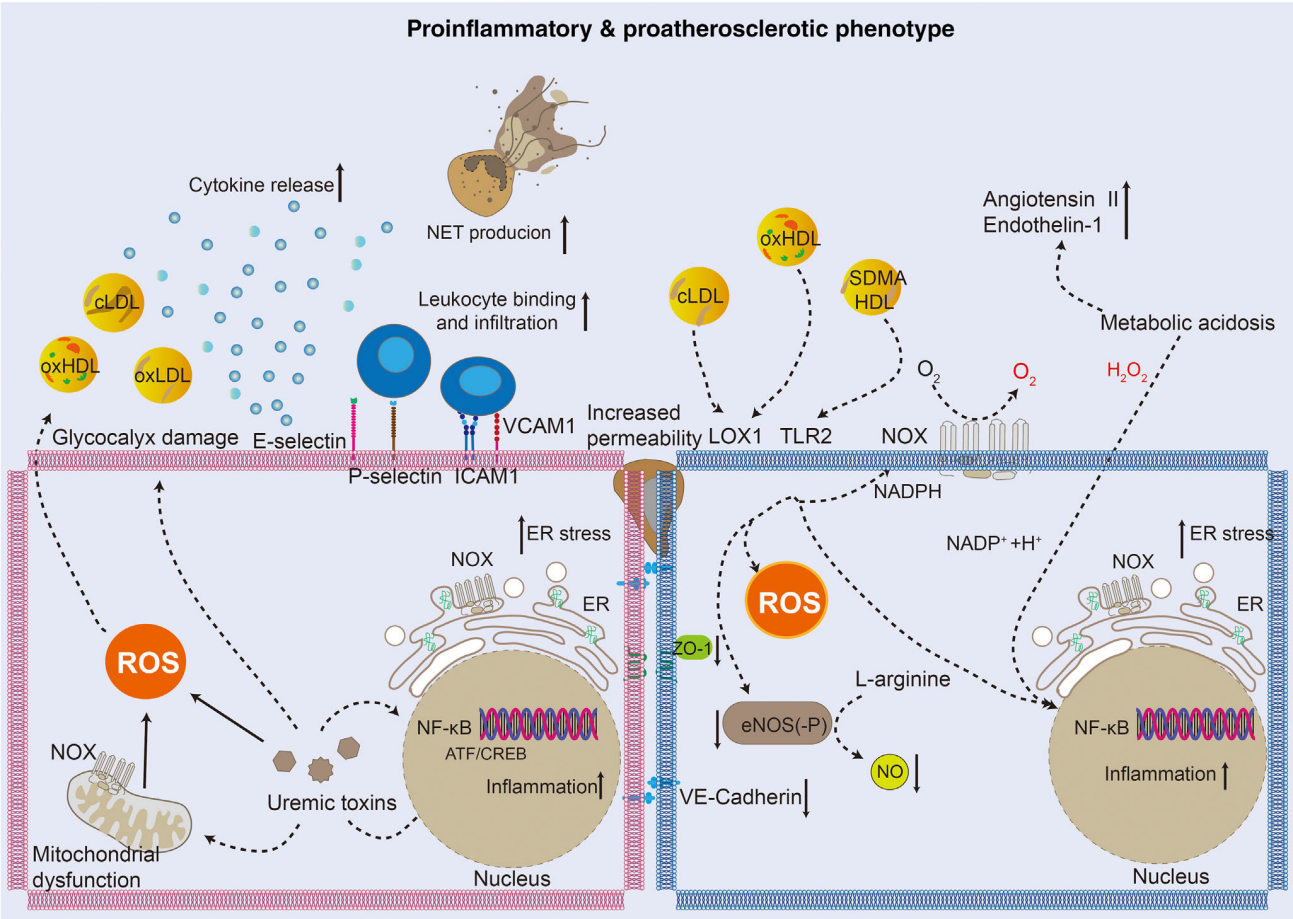
EC exhibits proinflammatory and proatherosclerotic phenotypes upon inflammation and atherosclerosis conditions. cLDL, carbamylated low‐density lipoprotein; ATF, activating transcription factor; CREB, cyclic adenosine monophosphate response element‐binding protein; eNOS, endothelial nitric oxide synthase; ER, endoplasmic reticulum; ICAM1, intercellular adhesion molecule 1; LOX‐1, lectin‐type oxidized low‐density lipoprotein receptor 1; NADPH, nicotinamide adenine dinucleotide phosphate; NET, neutrophil extracellular trap; NO, nitric oxide; NF‐κB, nuclear factor kappa‐light‐chain enhancer of activated B cells; oxHDL, oxidized high‐density lipoprotein; oxLDL, oxidized low‐density lipoprotein; TLR2, toll‐like receptor 2; VCAM‐1, vascular cell adhesion molecule 1; SDMA, symmetric dimethylarginine; ZO‐1, zonula occludens‐1.

Endothelial dysfunction occurs due to perturbations in oxidative stress, inflammation, and pathophysiological shear stress, leading to a reduction in antithrombotic and anti‐inflammatory functions, as well as degradation of the glycocalyx. Consequently, the dysfunctional EC adopts a proinflammatory and prothrombotic phenotype (Figure 3), characterized by decreased production of prostacyclin and NO, and increased release of proinflammatory and prothrombotic molecules such as C‐C motif chemokine ligand 2 and von Willebrand factor (VWF).^63^ Furthermore, the downregulation of thrombomodulin expression in EC leads to reduced activation of protein C, whereas the upregulation of tissue factor (TF) expression enhances coagulationactivation.^63^ Neutrophil extracellular traps are also implicated in endothelial dysfunction and atherothrombosis during the early stages of atherosclerosis.^64^

Oxidative stress and chronic inflammation contribute to the acceleration of EC aging, impacting their biological function and facilitating the development of various diseases. Senescent EC exhibit elevated secretion of osteopontin, interleukin‐15 (IL‐15), chemokine (C‐C motif) ligand 3, and growth differentiation factor 15 (GDF15).^65^ Mechanically, the barrier function of senescent EC is impaired, leading to the infiltration of immune cells and LDL‐cholesterol, thus promoting the occurrence and progression of atherosclerotic diseases.^66^, ^67^ Senescence of EC can be triggered by a range of atherosis‐related stimuli such as doxorubicin,^68^ TNF‐α,^69^ progerin,^70^ high glucose,^71^ and oxLDL.^72^

In response to LPS stimulation, NF‐κB increases expression of MCP‐1, IL‐6, IL‐1β, and TNF‐α in human peripheral blood mononuclear cells.^73^ Angiopoietin‐2 (ANG‐2), acting as an endothelial glycoprotein, promotes endothelial inflammation to facilitate angiogenesis and atherosclerosis. The p38 MAPK–cFOS pathway inhibits NO bioavailability in hyperinsulinemic insulin‐resistant subjects, resulting in an increase in Ang‐II expression and inflammation. The use of a neutralizing antibody to inhibit ANG‐2 can prevent hyperinsulinemic serum‐induced endothelial inflammation.^74^

Furthermore, mitochondrial dysfunction has been shown to trigger vascular inflammation, initiate endothelial dysfunction, and contribute to the development of atherosclerosis by generating excessive ROS. In human aortic endothelial cells, murine double minute 2 (MDM2) plays a role in regulating mitochondrial damage, thereby facilitating inflammation. Additionally, the suppression of MDM2 has been found to decrease the secretion of inflammatory cytokines IL‐6, TNF‐α, and IL‐1β, induced by oxLDL.^75^ Aside from NF‐κB and TLR4, Yes‐associated protein (YAP) and transcriptional coactivator with PDZ‐binding motif (TAZ) also mediate TNF‐α and LPS‐induced inflammatory responses.^76^, ^77^, ^78^ In addition, a Kruppel‐like factor 2 (KLF2)‐dependent target, TFEB, which is formed under laminar flow, suppresses the activity of IκB kinase in diabetic mice.^79^

A novel therapeutic approach for addressing endothelial dysfunction involves the inhibition of endothelial inflammation. Studies have demonstrated that KLF2 and KLF4, which serve as key regulators of vascular homeostasis, can suppress inflammation in EC in response to proinflammatory cytokines.^80^, ^81^ Recent research has indicated that the activation of G‐coupled‐protein receptor 81 can attenuate endothelial inflammation by partially restoring the downregulation of KLF2 induced by oscillatory shear stress, as well as by reducing the expression of MCP‐1 and VCAM1.^82^ Furthermore, the pharmaceutical solvent N‐methyl‐2‐pyrrolidone has been shown to ameliorate inflammation by activating KLF2.^82^

The study of endothelial inflammation has been a focus of extensive research due to its significant involvement in the initiation and advancement of CVD. Promising therapeutic approaches for treating CVD and endothelial dysfunction include NF‐κB inhibitors, YAP/TAZ inhibitors, and TLR4 antagonists. Ongoing research aims to uncover novel transcription factors that govern endothelial inflammation, highlighting the importance of identifying new drugs that target these inflammation‐associated transcription factors.

## 3 ENDOTHELIAL DYSFUNCTION IN SPECIFIC DISEASE CONDITIONS

**FIGURE 4:**
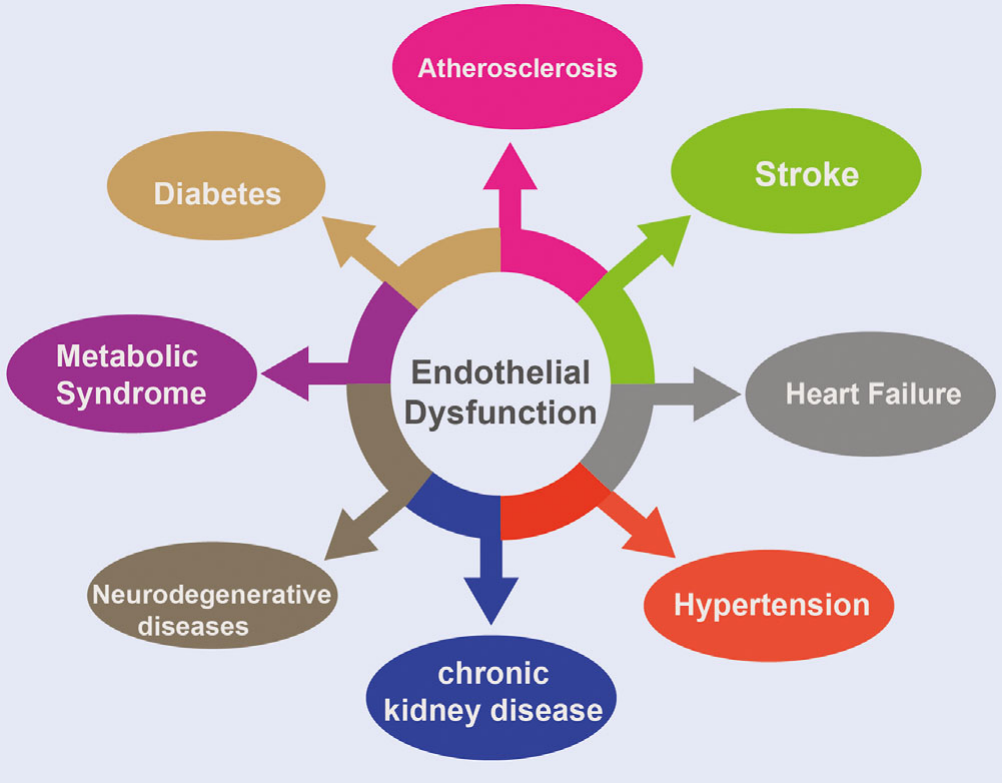
CVD and its complications are caused by endothelial dysfunction. Many disease conditions are caused by endothelial dysfunction due to imbalances in vasodilators and vasoconstrictors. Several major diseases such as atherosclerosis, diabetes, hypertension, stroke, peripheral arterial disease, CKD, and metabolic syndrome are associated with endothelial dysfunction.

Endothelial cell dysfunction is a significant contributor to multiple CVD and related complications (Figure 4). In the next section, we will provide an overview of the various diseases associated with endothelial dysfunction, with the aim of clarifying therapeutic targets and providing a basis for the development of future targeted drugs.

### 3.1 Endothelial function in HF

Sufficient levels of NO have been shown to possess antioxidant, anti‐inflammatory, and anticoagulant properties, as well as the ability to promote vasodilation.^61^ Normal endothelial function is essential for maintaining proper blood circulation in various organs, including the myocardium, coronary arteries, kidneys, and lungs.^83^ Endothelial dysfunction, characterized by decreased NO production, can result in compromised hemodynamics in cases of acute HF.^84^ Research indicates that a decrease in NO production mediated by eNOS is linked to impaired vascular tone, myocardial remodeling, and abnormal cell proliferation.^85^ Acute endothelial injury may occur in patients with acute HF, which is characterized by reduced NO production and endothelial dysfunction due to inflammation, excessive oxidative stress, and vasospasm following myocardial injury. This phenomenon is a prominent aspect of Takotsubo cardiomyopathy, accompanied by oxidative stress, excessive inflammatory response, and activation of the sympathetic nervous system.^84^ Studies have shown that pressure‐dependent NOS activation leads to acute endothelial cell hyperpermeability and rapid pulmonary edema by increasing NO and ROS in rats,^86^ suggesting that endothelial mechanotransduction plays a key role in the pathogenesis of acute HF.

Chronic HF is characterized by endothelial dysfunction, which is associated with adverse cardiovascular outcomes regardless of these verity or cause of HF.^87^, ^88^ Patients with chronic HF exhibit impaired peripheral tissue perfusion and vasoconstriction due to endothelial dysfunction.^89^ Thus, impaired tissue perfusion exacerbates preexisting vasoconstriction of coronary arteries or renal vessels.^85^ Increased levels of oxidative stress in patients with chronic HF can be attributed to the impaired functioning of the cellular antioxidant defense system, resulting from eNOS uncoupling and reduced NO production.^85^ Moreover, oxidative stress has been linked to disruption in Ca^2+^ homeostasis in diabetic cardiomyopathy.^90^ Furthermore, oxidative damage to cellular phospholipids has been shown to adversely impact microvascular and cardiomyocyte function in diabetic animal models.^91^ The decreased NO/ET‐1 ratio in chronic HF patients is correlated with persistent cardiac dysfunction, which can be reflected by echocardiographic measures, including left ventricular fraction shortening and left ventricular ejection fraction.^92^

With regard to myocardial fibrosis, in chronic HF, the transformation of endothelial cells into fibroblast‐like cells is known as endothelial‐to‐mesenchymal transition (EnMT).^93^ There is evidence that epigenetic regulation, including histone modification, DNA methylation, and the role of noncoding RNAs (long noncoding RNAs, miRNAs, and circular RNAs), is involved in EnMT processes in chronic HF.^93^ PI3K–AKT–eNOS–NO and HIF‐2α–Arg–NO axis are NO‐related pathways that regulate right ventricular and pulmonary vascular remodeling in rats of hypoxic pulmonary hypertension.^94^ According to a recent study in patients with HF with preserved ejection fraction suggests that impaired NO metabolism may be a key pathogenic determinant.^95^

### 3.2 Endothelial dysfunction in atherosclerosis

The significance of endothelial function in the development of atherosclerosis has been extensively documented. Endothelial dysfunction facilitates the infiltration of LDL into the subendothelial layer, leading to its accumulation and subsequent oxidation to ox‐LDL. This process triggers the expression of cell adhesion molecules, including VCAM1 and ICAM1, by EC, which in turn attract inflammatory immune cells into the sub‐endothelial space.^96^, ^97^ Various cardiovascular risk factors, including hyperglycemia, hyperlipidemia, aging, and smoking, further exacerbate immune cell migration, often resulting in chronic inflammation.

Hemodynamic alterations are a risk factor for the development of atherosclerosis due to their role in promoting endothelial dysfunction.^98^ Different shear stresses exerted on arterial walls play a crucial role in regulating various physiological processes, such as vascular tone, homeostasis, and vascular integrity.

Vascular segments experiencing low wall shear stress (WSS) or high oscillating WSS are particularly susceptible to atherosclerosis.^99^, ^100^ Disrupted WSS can lead to structural and functional damage to the vascular endothelium, ultimately triggering the proliferation and migration of VSMC and monocytes.^101^, ^102^ The mechanosensitive receptors on the endothelium are able to detect the magnitude and direction of WSS. Endothelial dysfunction at branch points and bifurcations is characterized by alterations in the expression levels of specific inflammation‐related proteins in the EC. For instance, the levels of eNOS, KLF2, and KLF4 are decreased in EC at vascular branching points, whereas the levels of inflammatory adhesion molecules such as P‐selectin, VCAM1, and ICAM1 are elevated.^103^ Because of special anatomical location, EC are constantly stimulated by many substances in the blood, including ROS, metabolites of glucose, lipids and amino acids, inflammatory cytokines, and immune cells. In regions where blood flow is disrupted, EC turnover is increased, leading to detrimental hemodynamic effects, whereas in areas with undisturbed blood flow, EC exhibit a prolonged lifespan.^104^

Aging is a significant risk factor for CVD and endothelial dysfunction. The aging process of EC serves as the initial stage in the development of age‐related diseases, ultimately leading to the occurrence and progression of endothelial dysfunction and other relateddiseases.^104^, ^105^ Furthermore, senescent endothelial cells exhibit diminished NO production and increased expression of NADPH oxidase and prostaglandin G/H synthase 2, commonly referred to as cyclooxygenase‐2, resulting in the activation, adhesion, and aggregation of platelets.^106^, ^107^, ^108^ Reduced production of NO promotes white blood cell adhesion and infiltration,^109^, ^110^ increases the proliferation and migration of VSMC,^106^, ^111^ and decreases delivery of O_2_ by red blood cells,^106^ collectively contributing to the advancement of atherosclerosis. Many molecules or pathways are involved in the pathophysiological changes of aging, including SIRT1,^112^ SIRT6,^113^ Klotho,^114^ and FGF21.^115^ Due to “inflammatory aging” of EC leading to endothelial dysfunction and CVD, drugs such asresveratrol,^116^ rapamycin,^117^ and chlorogenic acid^118^ are used to combat aging and improve age‐related diseases.

### 3.3 Endothelial dysfunction in hypertension

Pathogenic factors can contribute to changes in vascular resistance through various mechanisms, including the induction of abnormal vasoconstriction and dilation, as well as the promotion of proliferation and migration of VSMC. Endothelial dysfunction is considered as a significant indicator of alterations in vascular phenotype among individuals with hypertension.^119^ The reduction in NO bioavailability is a crucial factor in the pathophysiology of hypertension, alongside oxidative stress and vascular inflammation.^119^, ^120^ These pathogenic factors can contribute to changes in vascular resistance through various mechanisms, including the induction of abnormal vasoconstriction and dilation, as well as the promotion of proliferation and migration of VSMC.^119^ The age‐related increase in stiffness of large arteries is believed to be a key factor contributing to the increase in the prevalence of hypertension, whereas in younger individuals, elevated peripheral resistance is considered a major mechanism.^121^ In addition to NO, endothelium‐derived hyperpolarization (EDH) factors, including hydrogen sulfide, carbon monoxide, and eicosatetraenoic acid, K^+^, and C‐natriuretic peptide also play a crucial role in regulating vascular tone.^122^ Recent studies have shown that NO mainly mediates vasodilation of larger vessels (such as epicardial coronary arteries), whereas EDH mainly mediates vasodilation of small resistance vessels (such as coronary microvessels).^123^

ET‐1 is a potent endogenous vasoconstrictor synthesized by EC, VSMC, and macrophages. Its vasoconstrictive effects are mediated through the activation of ETA and ETB receptors. Prolonged upregulation of ET‐1 expression in mice has been associated with impaired endothelial function, sustained elevation in blood pressure, increased stiffness of small arteries, and early kidney injury. These detrimental effects were mitigated or reversed following a treatment with the ETA receptor antagonist, atrasentan for 2 weeks.^124^ Research has indicated that high salt intake significantly impacts endothelial function in rodent models.^125^

The endothelial cortex, located at 50–150 nm beneath the plasma membrane, serves as a significant target for the effects of salt.^126^ The stiffness of endothelial cortex plays a crucial role in cellular responses to both biochemical and physical stimuli. In normal physiological circumstances, the soft endothelial cortex is easy to deform, and the release of NO induced by shear stress is high. However, in the presence of high salt‐induced cortical stiffening, NO production is reduced, ultimately causing vascular constriction.^124^ High salt directly affects the phenotype of EC, resulting in endothelial cortex stiffening and reduced NO release. This can lead to increased blood pressure and hypertension complications, such as stroke, myocardial infarction, and CKD.^127^

### 3.4 Endothelial dysfunction in diabetes mellitus

Endothelial dysfunction has been implicated in both diabetic animal models and human patients.^127^ It is currently widely believed that excess ROS produced by mitochondrial and cytoplasmic oxidases (such as NADPH oxidase) in EC trigger the early stages of microvascular damage. Elevated levels of glucose or fatty acids can expedite this initial pathological process. Shah et al.^128^ have proposed a compelling hypothesis that EC respond to hyperglycemic stimulation by producing an abundance of ROS. This ROS‐induced DNA damage triggers the activation of DNA repair polymerase (ADP ribose polymerase 1), leading to ADP ribosylation and subsequent inhibition of cytoplasmic glyceraldehyde‐3‐phosphate dehydrogenase. This inhibition, in turn, hinders glycolysis reactions and promotes the accumulation of glycolytic intermediates. Subsequently, these effects increase the hexose flux by activating the sorbitol and glutamine‐fructose transaminase pathways, increase diacylglycerol by upregulating the concentration of triose phosphate, activate some protein kinase C (PKC) isomers (β, δ, and θ), and provide a substrate for the synthesis of methylglyoxal. Increased methylglyoxal levels and enhanced PKC activity can facilitate the activation of the NF‐κB inflammatory pathway, thereby contributing to the initiation of an injury response.^128^

In the context of the microvascular system in vivo, these injury processes affect the physiological function of EC (the most susceptible), VSMC, pericytes, and fibroblasts. The upregulation of nuclear factor erythroid 2‐related factor 2 (NRF‐2) serves as a stress response of vascular cells in response to excess ROS. Hyperglycemia results in an increase of NRF‐2 expression in ECs, whereas the protective effect of NRF‐2 knockout mice against hyperglycemia‐induced injury is diminished.^129^ These findings suggest that the balance between ROS and the antioxidant system plays a crucial role in modulating the extent of ROS‐induced microvascular damage. Significantly, elevated glucose levels have been shown to induce microvascular damage in the body over a relatively short period of time.^128^ Nevertheless, it is not clear why it takes decades or more for hyperglycemia to cause microvascular disease in humans. Evidently, there are numerous pathological mechanisms that may accelerate the development of microvascular complications have yet to be identified.

Currently, the pathogenesis of diabetic vascular complications mainly focuses on four pathways: the generation of advanced glycation end products (AGEs), the increased flux of the hexosamine pathway, the increased flux of the polyol pathway and the activation of PKC.^130^, ^131^ Nonenzymatic reactions involving monosaccharides such as glucose, glyceraldehyde, and fructose with amino groups of proteins, nucleic acids, and lipids can lead to the generation of AGEs.^132^, ^133^ Under hyperglycemia, the binding of AGEs to the AGE receptor (RAGE) on the cell surface triggers the production of ROS.^134^, ^135^, ^136^

AGEs bind to the RAGE on EC, leading to the activation of mitogen‐activated protein kinase (MAPK) and the upregulation of ET‐1 and lysine oxidase, resulting in a disruption of endothelial homeostasis.^136^ In individuals with type 2 diabetes mellitus (T2DM) and coronary atherosclerosis, there is a negative correlation between plasma levels of AGEs and endothelial function. In human coronary endothelial cells (HCAECs) AGEs induce oxidative stress by activating p38 and ERK1/2 pathways and reducing eNOS expression, ultimately causing endothelial dysfunction.^137^ In db/db mice, overexpression of peroxidase (PXDN) is observed along with impaired endothelium‐dependent dilation.^138^ The upregulation of PXDN and hypochlorous acid (HOCl) expression in HUVECs is facilitated by AGE–RAGE signaling. PXDN‐derived HOCl contributes to diabetic endothelial dysfunction by suppressing Akt–eNOS phosphorylation and NO production.^138^ Elevated levels of EMPs are detected in the serum of individuals with T2DM. AGE/RAGE signaling stimulates EMP release via the NOX–ROS pathway, thereby playing a significant role in the pathogenesis of diabetic vascular complications.^139^

### 3.5 Endothelial dysfunction in CKD

CKD is linked to impaired peripheral vascular and coronary microvascular function, as well as a reduction in the structural and functional density of capillaries with the progression of CKD.^140^, ^141^, ^142^ Nephrosclerosis, the predominant form of CKD, is characterized by vascular endothelial system disruption in the kidney,^143^ and endothelial dysfunction is prevalent in patients with CKD.^144^ It is widely accepted that blood pressure and flow play a crucial role in determining renal function and glomerular filtration rate, thus endothelial dysfunction‐induced blood flow impairments have a direct impact on renal function.

Elevated levels of C‐reactive protein (CRP), TNF‐α, and IL‐6 are commonly observed in the circulating blood of patients with CKD.^145^, ^146^, ^147^ As CKD progresses, activated EC releases soluble adhesion molecules such as vWF, matrix metalloproteinases (MMP), ICAM1, and VCAM1. The level of vWF is increased in both predialysis CKD patients and those undergoing dialysis.^148^

The rats in the CKD model exhibited a reduction in endothelium‐dependentrelaxation.^149^ In a rat model of proteinuric kidney disease, there is an increase in endothelial permeability of thoracic aorta and microvascular permeability, accompanied by the absence of the endothelial surface layer.^150^ Additionally, five out of sic nephrectomized rats displayed significant glycocalyx damage, as evidenced by decreased arterial glycocalyx thickness and elevated plasma levels of the glycocalyx component syndecan‐1.^151^ Endothelial dysfunction has been observed in a mouse model of CKD, as evidenced by a marked decrease in glycocalyx thickness and density, upregulation of glycocalyx components, impaired acetylcholine‐induced endothelium‐dependent relaxation, and elevated expression of ICAM1 and VCAM1 in EC.^152^, ^153^, ^154^ Furthermore, Ehling et al.^155^ discovered that renal impairment led to reduced vessel diameter, as well as increased vessel tortuosity in three mouse models of advancing kidney disease and renal fibrosis, as determined through microcomputed tomography imaging of the preglomerular arteries. In ApoE^−/−^ mice, CKD induces a higher plaque burden, suggesting a higher risk of atherosclerosis in CKD conditions.^152^ Various pathophysiological mechanisms have been implicated in the development of endothelial dysfunction in CKD, including chronic low‐grade inflammation, oxidative stress, accumulation of uremic toxins, metabolic acidosis, posttranslational modifications, and hyperactivation of the sympathetic nerve. In the context of renal injury, chronic low‐grade inflammation triggers the release of proinflammatory cytokines and chemokines from resident renal cells, promoting extracellular matrix (ECM) deposition and ultimately contributing to tubulointerstitial fibrosis. Consequently, chronic low‐grade inflammation emerges as a key driver of CKD progression.^156^ Moreover, in individuals with CKD, diminished renal function results in a systemic uremic state that induces chronic low‐grade inflammation through the upregulation and secretion of proinflammatory factors.^157^ This inflammatory response in CKD is characterized by elevated levels of systemic proinflammatory cytokines such as IL‐1β, IL‐6, TNF‐α, and highly sensitive CRP (hsCRP), which are accumulated in the blood due to enhanced release and impaired clearance by the kidneys.^158^, ^159^ The rat model of CKD exhibited elevated systemic AGEs levels, leading to increased endothelial NADPH oxidase‐2 (NOX‐2), cytoplasmic, and mitochondrial ROS levels, while simultaneously decreasing mitochondrial SIRT3 levels, which are crucial in mitochondrial ROS generation.^160^ Furthermore, CKD‐induced posttranslational modification of LDL and HDL resulted in endothelial eNOS uncoupling, leading to enhanced ROS production in EC.^161^, ^162^, ^163^ These modifications of lipoprotein particles in CKD may promote LDL to become more harmful and convert HDL from protective to detrimental lipoprotein particles.

Uremic toxins, which are organic or inorganic substances that accumulate in circulation as a result of diminished renal function or increased production, are widely recognized for their detrimental effects on the body. In vitro, uremic serum collected from CKD patients reduced the height of endothelial glycocalyx and increased the stiffness of both the glycocalyx and the endothelial cortex. These effects, along with reductions in eNOS and NO levels induced by uremic serum, can be mitigated by inhibiting the mineralocorticoid receptor and its downstream target.^164^ Uremic toxins, including p‐formylsulfate, cyanate, indolyl sulfate, asymmetric dimethylarginine, AGEs, and uric acid, have been documented to elicit oxidative stress and inflammation, as well as facilitate leukocyte adhesion to endothelium.^165^, ^166^ Furthermore, these toxins have been shown to inhibit EC proliferation and induce cell death.^165^, ^167^ Additionally, cyanate has been found to enhance the thrombogenic effect of EC by upregulating the expression of TF and plasminogen activator inhibitor‐1.^165^ Mechanistically, uremic toxins activate inflammatory signaling pathways and oxidative stress in EC, including cAMP response element‐binding protein(CREB)/AMP‐dependent transcription factor 1 (ATF1), MAPK/NF‐κB, RAGE, and aryl hydrocarbon receptor signaling pathways.^165^, ^166^ In HUVECs stimulated by uremic media, disruptions in VE–cadherin interactions and F‐actin reorganization can be observed.^168^ These findings suggest that uremic toxins play a significant role in the development of CVD in patients with CKD. Meta‐analyses have demonstrated an association between the uremic toxin p‐cresylsulfate and cardiovascular risk in CKD patients.^169^

### 3.6 Endothelial dysfunction in neurodegenerative diseases

The cerebrovascular system plays a crucial role in the regulation of cerebral blood flow to ensure the stability and balance of the central nervous system (CNS). Disruption of the BBB is currently recognized as an early indicator of neurodegenerative disorders, including Alzheimer's disease (AD), amyotrophic lateral sclerosis, and Parkinson's disease.^170^, ^171^

The BBB serves as a bridge between the CNS and the peripheral circulation, with distinct structural differences observed in microvessels within the BBB compared with peripheral vessels. The microvessels within the BBB are characterized by the presence of specific transporters, tight junction (TJ) proteins, and ion channels, as well as a single layer of EC and abundant mitochondria.^172^ The BBB serves to protect the CNS from pathogens, regulate the transport of essential nutrients, O_2_, and metabolic waste, maintain ion balance within the CNS, and prevent the entry of harmful substances. Under normal physiological conditions, macromolecules are unable to pass through the BBB. It is widely recognized that the integrity of the BBB is compromised in the context of aging and neurodegenerative diseases.^173^, ^174^

AD is a neurodegenerative condition characterized by significant cognitive impairment. Pathologically, AD is marked by the accumulation of β‐amyloid protein (Aβ) and tau neurofibrillary tangles.^172^ The breakdown of the BBB in AD is believed to be caused by the deposition of Aβ in the vascular system, leading to neurotoxicity and inflammation.^175^ This BBB dysfunction is closely associated with cognitive decline in individuals with AD.^176^ Soluble PDGFR‐β (sPDGFR‐β), which is highly expressed in pericytes surrounding capillaries in brain, has been identified as a potential biomarker for BBB dysfunction in cerebrospinal fluid.^176^ Significantly, individuals with cognitive impairment demonstrate elevated levels of PDGFR‐β, whereas the accumulation of Aβ and tau proteins does not exhibit a substantial increase.^176^ These results suggest that disruption of the BBB could serve as a potential biomarker of cognitive decline and may manifest in the early stages of AD. Recent research indicates that apolipoprotein E (APOE4), a well‐established genetic predisposition for AD, may play a role in BBB dysfunction.^177^ Individuals carrying the APOE4 (ε3/ε4 or ε4/ε4 allele) exhibit distinct patterns of BBB disruption compared with noncarriers, particularly in the hippocampus and medial temporal lobe. The extent of cognitive decline in APOE4 carriers is closely linked to markers of BBB destruction, such as MMP‐9, which plays a direct role in BBB destruction.^177^ These results indicate that BBB dysfunction may be a contributing factor to cognitive decline in individuals with AD. Inflammation within the CNS frequently results in significant impairment of BBB function and integrity. For instance, in cerebral amyloid angiopathy, the permeability of the BBB is increased as a result of diminished expression of TJ proteins like claudin‐5, ZO‐1, and occludin.^178^ Amyloid accumulation‐induced inflammation serves as the primary pathological marker.^178^ Furthermore, the activation of microglia triggers BBB dysfunction, resulting in the depletion of TJ proteins in EC and pericytes, as well as an elevated release of cytokines and chemokines (IL‐6 and MCP‐1).^179^

The ECM component within the CNS is crucial for the regulation of the BBB structure and function. Although ECM is traditionally recognized for its role in maintaining vascular system stability, numerous ECM components facilitate cell‐to‐matrix and cell‐to‐cell interactions. Specifically, the laminin family, predominantly found in the basal membrane, interacts with EC through integrins and subsequently binds to other matrix components such as agrin and perlecan.^180^ MMPs are instrumental in governing ECM remodeling. In numerous neurodegenerative disorders, there is an upregulation in the expression or activity of MMPs, which may be closely related to BBB impairment. Specifically, in rats experiencing epileptic seizures, there was a notable elevation in the expression and activity of MMP‐2 and MMP‐9.^181^ Furthermore, Overexpression of MMP‐9 has been shown to promote the development of epilepsy following traumatic brain injury.^182^

An increasing body of research is exploring therapeutic interventions aimed at enhancing the integrity and functionality of the BBB. Donepezil, anacetylcholinesterase inhibitor known for its efficacy in improving psychiatric symptoms and cognitive function in AD,^183^ has been demonstrated to mitigate BBB damage through upregulation of claudin‐5 expression.^184^ Moreover, modulation of MMP activity, a key indicator in the development of epilepsy, has shown promising therapeutic benefits. For example, the MMP inhibitor IPR‐179 exhibits antiepileptogenic and antiseizure properties in animal models of epilepsy, as well as the ability to attenuate epilepsy‐induced cognitive decline.^185^

## 4 CLINICAL IMPLICATIONS OF ENDOTHELIAL DYSFUNCTION

### 4.1 Endothelial dysfunction as a biomarker for predicting cardiovascular risk

Endothelial dysfunction has been identified as the primary factor in the development of numerous diseases, including hyperlipidemia, hypertension, diabetes, and CKD.^10^, ^186^ ANGPTL2, an inflammatory mediator, has been shown to exacerbate vascular inflammation and atherosclerosis.^187^ In a study by Horio et al.,^187^ it was discovered that the expression of ANGPTL2 in the aortic tissue of ApoE^−/−^ mice was positively associated with the development of atherosclerosis. In a study conducted by Hata et al.,^188^ serum levels of ANGPTL2 were analyzed in a cohort of 3005 Japanese individuals aged ≥40 years without a history of CVD. The results indicated that higher serum ANGPTL2 concentrations were correlated with an increase in age‐ and sex‐adjusted incidence rates of CVD.^188^

Endocan, a marker of endothelial dysfunction primarily released by activated endothelium, is found to be elevated in patients with hypertension.^189^ It is encoded by the gene endothelial cell‐specific molecule‐1 (ESM1).^190^ The secretion of endocan is regulated by proinflammatory cytokines such as IL‐αand TNF‐α.^190^ Upregulation of endocan expression has been shown to promote the formation of atherosclerotic plaques by inducing the expression of ICAM1 and VCAM1^189^, suggesting a potential link between elevated endocan levels and atherosclerotic plaque formation in individuals with hypertension. Several studies have demonstrated a correlation between high levels of CRP and carotid intima‐media thickness.^189^

In individuals with pulmonary hypertension, elevated levels of human epidermal growth factor receptor 3 (ErbB3), also known as HER3, were observed in the lungs, serum, and distal pulmonary arteries compared with healthy controls.^191^ There is evidence of endothelial dysfunction in diabetic patients based on elevated vWF plasma levels, decreased prostacyclin and plasminogen activator levels.^192^ A clinical investigation involving 32 women with type 1 diabetes and 25 healthy women revealed that endogenous estradiol exacerbates oxidative stress and contributes to endothelial dysfunction in diabetic females. Additionally, it appears that oral hormonal birth control may improve endothelial function in patients with type 1 diabetes.^193^

EMPs, which are small vesicles ranging from 0.1 to 1.0 mm in size, are found to be present at low levels in the plasma of healthy individuals, but elevate in cases of acute or chronic CVD associated with endothelial dysfunction, such as atherosclerosis, sepsis, diabetes mellitus, and acute coronary syndrome.^194^ In accordance with this, a research study involving 151 severely obese women and 60 lean controls has demonstrated that obese individuals exhibit elevated levels of EMPs compared with individuals of normal healthy weight.^195^ Additionally, a significant increase in CD62^+^ and CD144^+^ microparticles was observed in blood samples from 29 participants exposed to hypoxic conditions.^196^ Factors such as high glucose, Ang II, and ox‐LDL are known to be underlying stimuli of CVD, which can impact EC function and EMP production. EC‐derived EVs isolated from electronic‐cigarette users have been found to decrease NO production and increase ET‐1 production in human cerebral microvascular endothelial cells.^197^ Similarly, it has been shown that exposure of EC to ER stress leads to the secretion of EVs, which in turn inhibit angiogenesis of HUVECs and reduce NO release.^198^ Additionally, research has indicated that CD31^+^/CD42b^−^ endothelial EVs, derived from the plasma of individuals with Perthes disease, contribute to endothelial dysfunction and apoptosis of HUVECs.^199^ Furthermore, a recent study involving 76 participants designated as current smokers and nonsmokers revealed that a short session of tobacco smoking results in an elevated release of EC‐derived EVs and a decline in endothelial function.^200^

Recent research indicates that circulating miRNAs have the potential to function as biomarkers for a range of diseases. Various bodily fluids, including saliva, blood, tears, urine, and breastmilk, contain miRNAs that have shown utility as biomarkers for endothelial dysfunction and associated conditions.^201^ Furthermore, circulating miRNAs have been proposed as specific, sensitive, and noninvasive markers for the monitoring and early detection of diabetes (both type 1 and type 2) and the associated microvascular and macrovascular complications.^202^, ^203^, ^204^ A recent report indicates that exosomes isolated from individuals with diabetes type 2 exhibit elevated levels of miR‐20b‐5p.^205^ Furthermore, Katayama et al.^206^ observed significantly higher concentrations of exosome‐enriched miR‐20b‐5p in serum of type 2 diabetic males compared with healthy controls. Additionally, it was discovered that exosomes derived from mouse brain endothelial cells contain higher levels of miR‐126 than those from VSMC and marrow stromal cells.^207^

### 4.2 Therapeutic strategies targeting endothelial dysfunction

**FIGURE 5:**
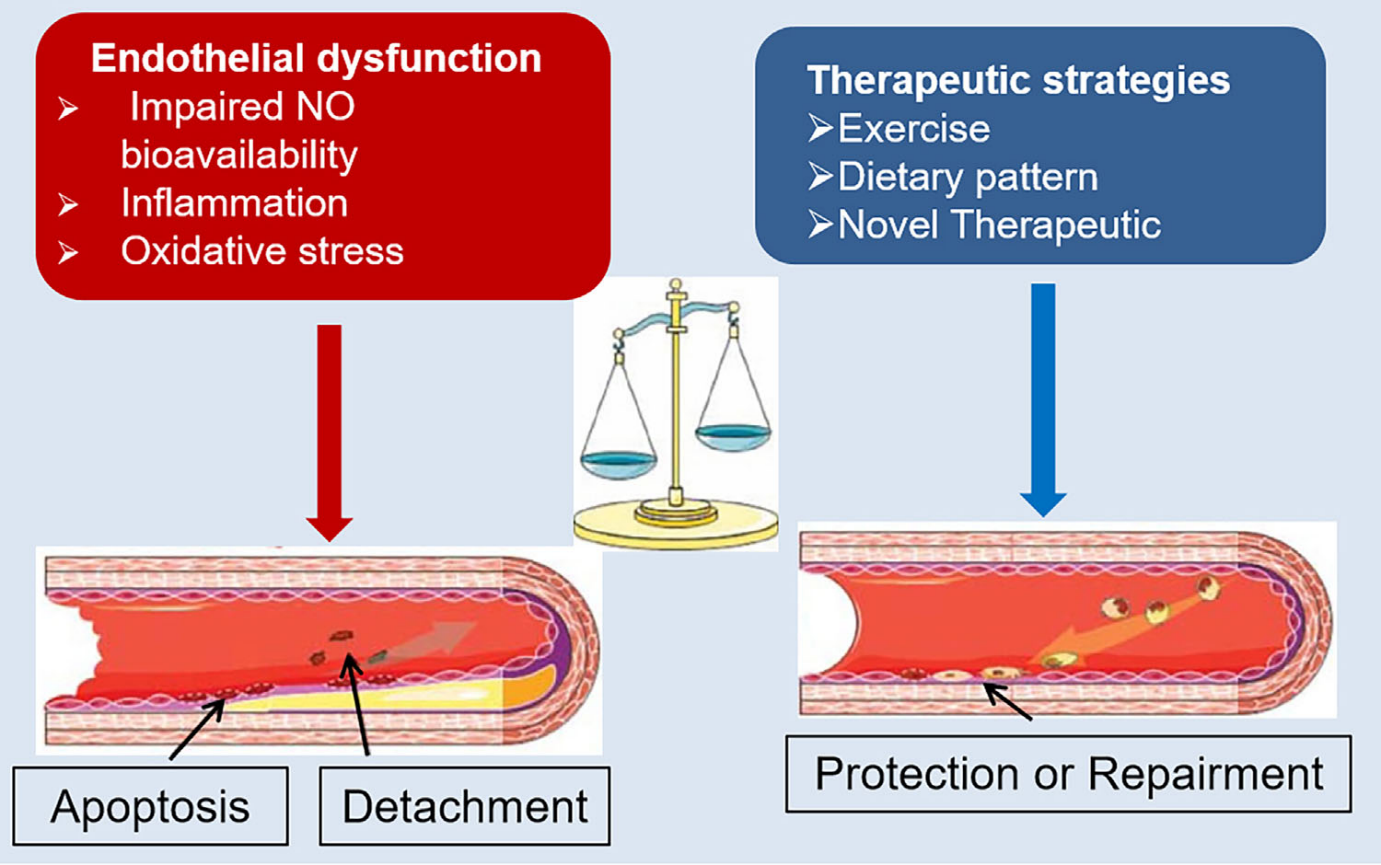
Endothelial dysfunction is caused by many factors and can be improved by several therapeutic strategies.

The initial manifestation of vascular impairment in CVD is the onset of endothelial dysfunction, highlighting the potential clinical significance of therapeutic interventions targeting this condition. Such interventions should encompass lifestyle modifications, including regular physical activity, smoking cessation, healthy eating, and the management of risk factors associated with atherosclerosis, such as diabetes, hypertension, dyslipidemia, and obesity (Figure 5). Enhancing endothelial function has been shown to impede the progression of established CVD. A large number of preclinical and clinical studies have attempted to find further evidence for endothelial function management. We summarized some of the major clinical studies of endothelial function therapy in Table 1.

**TABLE 1:** Major preclinical and clinical trials of therapeutic strategies on endothelial function.

Study name	Type of study	Interventions	Detecting method	NCT number
Vascular effects of ACEI and statins in adolescents with type 1 diabetes^208^	Randomized controlled trial	ACEI (Quinapril), statin	FMD,RHI, carotid‐femoral PWV	NCT01581476
The effect of IMST in blood pressure and endothelial function^209^	Randomized controlled trial	IMST	FMD, carotid‐femoral PWV	NCT03266510
The effect of mediterranean diet on endothelial function in patients with CHD^210^	Randomized controlled trial	Mediterranean diet	FMD	NCT00924937
Impact of intensive lifestyle treatment on endothelial and vascular function^211^	Randomized controlled trial	Diet plus exercise with monthly visits	ELISA, ultrasound	–
The effect of wild blueberry (poly)phenols on vascular function^212^	Randomized controlled trial	Blueberry (poly)phenols intake	FMD, PWV	NCT04084457
The effect ofBH_4_ on endothelial function in patients with cystic fibrosis^213^	Randomized controlled trial	Oral BH4	FMD	–
The effect of Ramipril on endothelial function in patients with SLE^214^	Randomized controlled trial	Ramipril	FMD	NCT03979976
Effects of alirocumab on endothelialfunction^215^	Randomized controlled trial	Alirocumab	FMD, IVUS, OCT	–
Effects of olmesartan and amlodipine on endothelial function, and vascular inflammation^216^	Randomized controlled trial	Olmesartan, amlodipine	FMD	–

Abbreviations: BH4, tetrahydrobiopterin; carotid‐femoral PWV, carotid‐femoral pulse wave velocity; CHD, coronary heart disease; FMD, flow‐mediated dilation; IMST, high‐resistance inspiratory muscle strength training; IVUS, intracoronary intravascular ultrasound; OCT, optical coherence tomography; RHI, reactive hyperemia index; SLE, systemic lupus erythematosus.

The impact of physical activity on endothelial function has been extensively investigated, particularly in populations with increased cardiovascular risk.^217^, ^218^ Exercise has been shown to effectively enhance endothelial function and decrease cardiovascular risk in postmenopausal women, older adults, and individuals with dyslipidemia. Additionally, exercise training has been found to ameliorate endothelial dysfunction in patients with HF and coronary artery disease.^219^ Weight loss has been shown to mitigate endothelial dysfunction in overweight individuals.^220^ Mechanically, exercise prevents coronary endothelial cell senescence through FUNDC1‐dependent mitochondrial autophagy and protects against myocardial ischemia/reperfusion injury in mice.^221^

It is well known that poor dietary quality is associated with endothelial dysfunction diseases. Recommendations outlined in the “2017 AHA presidential advisory on diet” and the “2015 to 2020 Dietary Guidelines for Americans” emphasize the importance of reducing saturated fat intake and increasing consumption of unsaturated fats (especially polyunsaturated fat), in conjunction with adopting a Mediterranean‐style eating pattern.^222^, ^223^ Research has shown that adherence to Mediterranean diets is associated with decreased risks of all‐cause mortality, CVD, and type 2 diabetes.^210^, ^224^, ^225^ The Mediterranean diet is distinguished by its emphasis on the consumption of fruits, vegetables, nuts, legumes, seafood, whole grains, extra virgin fish, olive oil, and a moderate amount of red wine, while limiting intake of red meat and sugar.^226^ This dietary pattern is rich in bioactive components such as fiber, polyphenols, phytosterols, monounsaturated and polyunsaturated fats, as well as minerals and vitamins.^224^, ^226^ These components contribute to its lipid‐lowering, antioxidative, insulin‐sensitizing, anti‐inflammatory, antibacterial properties, and antithrombotic properties.^224^, ^226^

Patients with modifiable risk factors (diabetes, dyslipidemia, hypertension) and nonmodifiable conditions (sex, menopause, and aging) can receive pharmacological endothelial therapy to reduce and prevent endothelial dysfunction. Medications targeting underlying risk factors associated with atherosclerotic complications have demonstrated efficacy in restoring endothelial function and reducing the incidence of cardiovascular disorders in certain instances.

The effects of angiotensin‐converting enzyme inhibitors (ACEI) extend beyond their primary role in inhibiting the renin–angiotensin system, as they also diminish the inactivation of bradykinin, thereby promoting the release of NO.^227^ ACEI also reduces myocardial ischemia by preventing pathological vasoconstriction of coronary arteries.^228^ In a parallel randomized controlled trial and observational cohort study, the researchers investigated the effects of ACEI on adolescents at high risk of developing adverse cardio‐renal outcomes. It was shown that ACEI can improve endothelial function in adolescent high‐risk adolescents.^208^ In a clinical trial, 37 female patients with systemic lupus erythematosus (SLE) had no cardiovascular risk factors were randomly assigned to two groups: 19 received ramipril treatment for a duration of 12 weeks, whereas 18 did not. The findings indicated that ramipril treatment led to improvements in endothelial function and an increase in EPC numbers in SLE patients without cardiovascular risk factors. These results suggest that ACEI may offer additional benefits beyond blood pressure reduction.^208^ Angiotensin II receptor blockers (ARBs) have been shown to effectively reduce oxidative stress induced by AngII, thereby enhancing endothelial NO bioavailability in mice.^229^ Sartans have been identified as a viable treatment option for patients experiencing ACEI‐associated angioedema due to their ability to decrease levels of VEGF‐A, VEGF‐C, and sPLA2 in plasma.^230^ The irbesartan and lipoic acid in endothelial dysfunction study found that the ARB (irbesartan) can enhance endothelial function and reduce inflammation.^231^ Among third‐generation β‐blockers, nebilolol stands out as a highly selective receptor blocker that uniquely stimulates the production of NO. This distinctive characteristic of nebilolol suggests its potential to alleviate endothelial dysfunction by activating adrenergic receptors in the heart.^232^

Several studies have demonstrated that statins exhibit favorable effects on endothelial function, likely attributed to their antioxidant and anti‐inflammatory properties rather than their lipid‐lowering effects.^233^, ^234^ A meta‐analysis and systematic review of randomized controlled trials further support the notion that statin treatment is associated with notable enhancements in endothelial function of both coronary and peripheral arteries. It is uncertain whether improvements in endothelial function independently reduce vascular risk in lipid‐lowering therapy, and whether evaluation of endothelial function helps identify patients who need more aggressive lipid lowering.^235^ Furthermore, patients with arterial hypertension may benefit from statin therapy regardless of their plasma lipid levels or blood pressure.^236^ Additionally, evidence suggests that statins can effectively reduce endothelial dysfunction, inflammation, and oxidative stress in a mouse model of Kawasaki disease‐like vasculitis.^237^

Recently, several new pharmacological interventions have been shown to improve endothelial function. Estrogen can improve endothelial function by reducing ROS and increasing NO.^193^ A meta‐analysis of 22 studies with a total of 1472 patients showed that allopurinol significantly improved flow‐mediated dilation (FMD) compared with controls.^238^ It appears that alpha‐lipoic increases NO levels and reduces endothelial inflammation and oxidative stress of EC.^239^ Traditional Chinese medicine, particularly Chinese herbal monomer extracts, shows significant potential in protecting endothelial function. Resveratrol has been found to enhance total antioxidant ability and reduce levels of endothelial dysfunction biomarkers, such as vWF, ICAM‐1, and caspase 3 in endothelial cells and umbilical arteries from patients with preeclampsia.^240^ Astragalus polysaccharide has been shown to improve vascular endothelial dysfunction in diabetes by promoting macrophage polarization to M2 through activating Nrf2/HO‐1 pathway.^241^ Additionally, catalpol has been demonstrated to enhance VEGF–PI3K/AKT and VEGF/MEK signaling, improving impaired neurovascular function in rats with ischemic stroke.^242^

## 5 CLINICAL ASSESSMENT OF ENDOTHELIAL DYSFUNCTION

Endothelial function in the epicardial coronary arteries, peripheral conduit arteries, coronary or peripheral microvasculature can be assessed through invasive or noninvasive methods, including FMD, pulse wave velocity measurement, and venous occlusion plethysmography (Table 2).^243^ The assessment of coronary endothelial function through invasive quantitative angiography, which detects luminal changes in response to vasoactive stimuli that stimulate NO release from EC, remains a key focus in the study of vasoreactivity in the vascular system. Angiography of the epicardial arterial dimension is often performed with an intracoronary injection of acetylcholine.^244^ Other agents such as papaverine, bradykinin, and substance P are also utilized in endothelial‐dependent vasomotor testing.^245^ Invasive methods for diagnosing endothelial dysfunction have been reported in several cardiometabolic disease states and have been associated with the development of atherosclerosis.^246^ The arterial brachial FMD is the single most widely used noninvasive tool for detecting endothelial dysfunction.^119^ Furthermore, FMD can be used to stratify low, medium, and high risk populations for future cardiometabolic disease. Additional assessments for evaluating endothelial dysfunction involve measuring biomarkers in the blood that indicate harmful events in the heart and vessels, like the release of cytokines linked to inflammation (IL‐6 and IL‐1β). To complement established clinical tools for diagnosing arterial brachial FMD, novel endothelial‐specific biomarkers must be identified.

**TABLE 2:** An overview of endothelium‐mediated vasoreactivity.

Clinical assessment of endothelium‐mediated vasoreactivity	
Invasive	Noninvasive
Arterial infusion of endothelium‐dependent vasodilators (serotonin, bradykinin, acetylcholine, etc.)^247^	Reactive hyperemia after an interval of blood flow occlusion (FMD)^119^, ^247^
**Epicardial coronary arteries** Quantitative coronary angiograph or intravascular ultrasound **Coronary microvasculature** Quantification of CFR = ration of max. CBF upon endothelium‐dependent vasodilation to CBF at rest **Perivascular microvasculature** Venous occlusion plethysmography of forearm circulation	**Perivascular conduit arteries (gold standard)** Ultrasound analysis of FMD of brachial artery after an initial brachial artery occlusion with a blood pressure cuff **Perivascular microvasculature** Digital PAT or finger plethysmography, recording arterial pulsative volume changes in the fingertip following an initial artery occlusion

Abbreviations: CBF, coronary blood flow; CFR, coronary flow reserve; FMD, flow‐mediated dilation; PAT, peripheral arterial applanation tonometry; PAT, peripheral arterial tonometry.

Research on systemic biomarkers and their clinical application has increased exponentially over the last few decades.^248^ The discovery of systemic biomarkers provides valuable insights into the pathophysiology of atherosclerosis and the development of new therapies. Inflammation and endothelial dysfunction are key factors involved in the progression of CAD.^249^, ^250^

As mentioned before, numerous inflammation‐related factors have been identified as potential biomarkers for the early detection of CAD. The use of acute‐phase proteins, adhesion molecules, cytokines, and microparticles as markers of endothelial dysfunction and inflammation has been extensively studied in clinical studies. The process of inflammation and atherosclerosis has been extensively linked to cytokines, which are pleiotropic proteins. Proinflammatory cytokines play a crucial role as inflammatory mediators, promoting inflammation, upregulating adhesion molecules in EC, and contributing to endothelial injury. Endothelial dysfunction is exacerbated by proinflammatory cytokines such as TNF‐α, IL‐6, IL‐8, and IL‐18, which activate the inflammatory response, induce adhesion molecules like P‐selectin and E‐selectin, and intensify the inflammatory response. The expression of P‐selectin, E‐selectin, ICAM1, and VCAM1 is upregulated in EC by inflammatory cytokines.^10^ These adhesion molecules are released by activated EC. There is mounting evidence suggesting that elevated levels of soluble E‐selectin, VCAM1, and/or ICAM1 in circulation are linked to the severity and complications of coronary artery disease.^251^ In spite of this, it should be noted that although adhesion molecules are also produced by platelets and leukocytes, they are not specific biomarkers for detecting endothelial damage.^252^, ^253^ Consequently, the diagnostic value of circulating levels of adhesion molecules is limited when used alone.

Monocytes have emerged as potential markers for assessing vascular dysfunction and cardiac disease.^254^, ^255^, ^256^ Elevated monocyte counts in individuals diagnosed with coronary artery disease were found to be correlated with an increased risk of cardiovascular events and impaired peripheral endothelial function.^254^ Analysis of CD16^+^ monocyte and macrophage subtypes in patients undergoing cardiac catheterization revealed a direct association with the severity of coronary artery disease and an inverse relationship with M2 macrophages, indicating the potential utility of macrophage and monocyte subtypes as biomarkers.^256^

Numerous studies have examined the roles of lipids, proteins, and other metabolites in endothelial dysfunction, as well as the potential value of these compounds in clinical settings. New molecular biology techniques and liquid biopsies have identified several new potential biomarkers associated with coronary artery disease, summarized in Table 3.

**TABLE 3:** Potential biomarkers of endothelial dysfunction and inflammation in CAD.

Biomarkers	Object of study	Relevance to CAD	References
ANGPTL8	Cohort of patients with CAD	There was a significant increase in serum ANGPTL8 levels in CAD patients. ANGPTL8 was independently associated with ICAM‐1 and TG in CAD patients.	^257^
Cyr61	Cohort of patients with CAD	Cyr61 levels in serum were higher in CAD patients than that of controls and were positively correlated with Gensini score and CRP levels.	^258^
CTRP9	Cohort of patients with CAD and T2DM	CTRP9 expression is elevated in patients with CAD and T2DM.	^259^
Irisin	Cohort of children and adolescents with T2DM, metabolic syndrome	T2DM and metabolic syndrome patients showed lower levels of Irisin than healthy controls. There was a negative correlation between Irisin levels and levels of sVCAM‐1, sICAM‐2, and MCP‐1 in children and adolescents.	^260^, ^261^, ^262^
Renalase	Patients with acute chest pain presenting to the emergency room for diagnostic workup, including PET scanning to identify CMD	Renalase serum levels were increased in patients with acute chest pain who presented with symptoms of CMD. Increased renalase levels may serve as a biomarker for CMD.	^263^
Sortilin	Cohort of patients with CAD	Significant decreases in sortilin levels were observed in statin groups.	^264^
PCSK9	Patient cohort suspected of having CAD	Low plasma PCSK9 levels are correlated with an unfavorable metabolic profile and diffuse nonobstructive coronary atherosclerosis.	^265^
Phosphatidylcholine and lysophosphatidylcholine	Cohort of patients with PAD and CAD	The levels of lysophosphatidylcholine and phosphatidylcholine in blood serum of patients with CAD and PAD were lower than those in controls.	^266^
lncRNA KCNQ1OT1, APOA1‐AS, and HIF1A‐AS2	Cohort of patients with CAD	ROC analysis demonstrated their suitability as biomarkers of CAD.	^267^
Circulating microRNA‐33	Cohort of patients with CAD	Compared with controls, patients with CAD express a higher level of microRNA‐33.	^268^
Circulating microRNA‐92a	Cohort of patients with CAD + T2DM	The increase in microRNA‐92a levels in T2DM patients was significantly associated with increased ACS risk. In addition, The increase in microRNA‐92a levels in T2DM patients was significantly associated with increased ACS risk.	^269^
Circulating microRNA‐331, 151‐3p	Cohort of patients with STEMI	Patients with STEMI exhibited higher levels of microRNAs‐331 and 151‐3p than those with stable angina and controls, suggesting that these miRNAs may be correlated with plaque rupture.	^270^
Serum exosomal microRNA‐126, 21, and PTEN	Cohort of patients with ACS	In patients with ACS, exosomal microRNA‐126, 21 and PTEN levels were higher than in controls. Exosomal microRNA‐126 levels were positively correlated with coronary artery stenosis severity.	^271^
Circulating microRNA‐145	Cohort of patients with ACS	microRNA‐145 expression was reduced in ACS patients as compared with controls. A correlation was found between microRNA‐145 levels and other markers of endothelial inflammation.	^272^
Circulating microRNA‐22	Cohort of patients with CSF	Patients with CSF have higher microRNA‐22 levels than those controls. MicroRNA‐22 is considered an appropriate biomarker for CSF.	^273^
microRNA signature	Cohort of patients with CSF	The expression levels of miR‐208a, miR‐133, miR‐17, miR‐206, miR‐223, miR‐326, miR‐29, and 155 in PBMCs of CSF patients were significantly higher than those of controls.	^274^
microRNA signature	Cohort of patients with CSF	There was a significantly decreased expression of microRNAs: miR‐21, miR‐15a, miR‐126, miR‐25, miR‐18a, and miR‐16 in SCF patients when compared with control patients.	^274^

Abbreviations: ANGPTL8, angiopoietin like 8; APOA1‐AS, APOA1 antisense RNA; CAD, coronary artery disease; CMD, coronary microvascular dysfunction; CRP, C reactive protein; CSF, coronary slow flow; CTRP9, C1 q/TNF‐related protein 9; Cyr61, cysteine‐rich protein 61; HIF1A‐AS2, HIF1A antisense RNA 2; KCNQ1OT1, KCNQ1 opposite strand/antisense transcript 1; MCP‐1, monocyte chemoattractant protein; PAD, lncRNA, long noncoding RNA; PCSK9, proprotein convertase subtilisin/kexin type 9; peripheral artery disease; PTEN, phosphataseandtensin homolog; sICAM‐2, soluble intercellular adhesion molecule 1; STEMI, ST‐segment elevation myocardial infraction; sVCAM‐1, soluble vascular cell adhesion molecule‐1; TG, triglycerides.

## 6 NOVEL RESEARCH FINDINGS AND FUTURE DIRECTIONS

### 6.1 Emerging therapies for improving endothelial function

It has been traditionally recognized that natural products are a valuable source of cardiovascular drugs because of their inherent antioxidant and anti‐inflammatory properties. The compound 1m‐6, a derivative of chalcone, has been found to inhibit the JAK/STAT3 pathway and activate the Nrf2/HO‐1 pathway in HUVECs, leading to the inhibition of TNF‐α‐induced upregulation of VCAM1 and ICAM1.^275^ It has been discovered that halofuginone, an antimalarial drug, effectively suppresses the LPS‐induced production of proinflammatory cytokines and ICAM1 expression.^276^ This inhibition is achieved through the suppression of NOX2‐dependent ROS production and the upregulation of KLF2 via ERK5 signaling. The findings suggest that halofuginone may have potential as a therapeutic agent for combating endothelial dysfunction in atherosclerosis.^276^ Further exploration through high‐throughput or high‐content drug screening may identify other naturally occurring bioactive compounds that can serve as templates for drug optimization.

In recent years, increasing evidence has indicated that DNA methylation and histone modification are key factors in the development of endothelial dysfunction and atherosclerosis. Several epigenetic drugs approved by the United States Food and Drug Administration, such as histone deacetylase inhibitors (suberoylanilide hydroxamic acid), DNA methyltransferase inhibitors,^277^ and bromodomain‐containing protein 4 inhibitors,^278^ have been shown to effectively mitigate endothelial dysfunction. The safety profiles of epigenetic drugs have been partially established through their clinical use in treating various cancers, suggesting potential suitability for use in patients with CVD. Further research is needed to validate the efficacy of these drugs in cardiovascular applications. Additionally, whether the combination of these epigenetic drugs with different mechanisms will result in additional benefits is also need to be explored.

### 6.2 Challenges and limitations in assessing endothelial function

Endothelial dysfunction is associated with complex molecular mechanisms. The application of single‐cell RNA sequencing (scRNA‐seq) has demonstrated efficacy in the identification and assessment of previously undiscovered cell populations and their dynamic, heterogeneous, and plastic nature.^279^, ^280^, ^281^ Additionally, scRNA‐seq has unveiled that EC are metabolically plastic and contributes to maintaining the homeostasis of various tissues.^282^, ^283^ Moreover, in specific contexts, scRNA‐seq also reveals the heterogeneity of EC. Recent discoveries from scATAC‐seq (single‐cell sequencing of transposase‐accessible chromatin)and scRNA‐seq of mouse carotid arteries subjected to partial ligation have demonstrated that disrupted flow stimulates EC to generate atheroprone characteristics.^284^ Using these advanced technologies, researchers are able to identify novel plaque‐resident cell types and novel factors influencing biological responses to flow changes.

The utilization of multiomics technologies and systems biology has facilitated the elucidation of the regulatory mechanisms governing endothelial translation, transcription, and chromatin. For instance, Linna‐Kuosmanen et al.^285^ employed a comprehensive approach encompassing chromosome conformation capture, microRNA‐sequencing, RNA‐sequencing, and chromatin immunoprecipitation sequencing to decipher Nrf2 transcriptional in EC exposed to oxidized phospholipids. This investigation revealed Nrf2 as a novel regulator of endothelial microRNAs, with miR‐100‐5p and miR‐21‐5p identified as hub miRNAs.

Although FMD is commonly used in clinical settings as a primary measure of endothelial function, there are various other factors contributing to endothelial dysfunction that may be present simultaneously or act as concurrent triggers. A recent study revealed that Lp(a) can induce endothelial glycolysis, increase endothelial inflammation, and promote leukocyte transendothelial migration.^286^ A similar crosstalk also exists between cell senescence and metabolism, indicating a complex network of interactions that perpetuate endothelial dysfunction and tissue damage and ultimately result in endothelial dysfunction‐related disorders.^287^ Despite the emergence of various treatments aimed at preventing endothelial dysfunction, a definitive therapeutic targeting this condition remains elusive. As such, investigations at the cellular and animal levels are essential for identifying specific targets associated with endothelial dysfunction that contribute to vascular inflammation. Furthermore, more precise animal model and clinical studies are necessary to establish a clear correlation between endothelial dysfunction and CVD.

## 7 CONCLUSION

EC are ubiquitous in the human body and play a crucial role in the maintenance of homeostasis. Various diseases, such as metabolic diseases, infectious diseases, and CVD, have been linked to endothelial dysfunction. This dysfunction can be attributed to a range of mechanisms, including oxidative stress, impaired vasodilation, cellular senescence, inflammation, cell death, and EndoMT, with reduced NO bioavailability being a key feature. The development of pharmacological agents with endothelium‐protective properties holds promise for improving patient outcomes. There are several potential areas for further research in the field, such as investigating the intricate pathology of endothelial dysfunction and the heterogeneity in CVD, applying systems biology and multiomics, exploring the interaction between various aspects of endothelial dysfunction, and identifying novel natural products with therapeutic potential for targeting endothelial dysfunction.

In summary, despite tremendous research progress over the past decades into endothelial function, our understanding of endothelial dysfunction remains incomplete. Since EC are the first‐line gatekeepers of vascular health in human blood vessel, it may be possible to preserve endothelial health and maintain vascular function through the discovery of endothelial protective genes or therapeutic drugs.

## AUTHOR CONTRIBUTIONS

Xia Wang contributed to writing the manuscript. Ben He revised the manuscript before submission. All authors approved the final version of the manuscript.

## CONFLICT OF INTEREST STATEMENT

The authors declare that they have no conflict of interest.

## ETHICS STATEMENT

No ethical approval was required for this study.

## Data Availability

Not applicable.
